# Influence of organizational atmosphere on current and future professional benefits among staff nurses: a cross-sectional study

**DOI:** 10.1186/s12912-026-04349-w

**Published:** 2026-02-12

**Authors:** Shaimaa Mohamed Araby Ebraheem, Asmaa Saber Ahmed Elborai, Marwa Abd Elrahman Gaber, Eman Hassan Mohamed Ali

**Affiliations:** 1 Nursing Administration Department, Faculty of Nursing, Benha University, Benha, Egypt; 2 Nursing Administration Department, Faculty of Nursing, Ain Shams University, Cairo, Egypt

**Keywords:** Organizational atmosphere, Current and perceived professional benefits, Staff nurses

## Abstract

**Background:**

Organizational atmosphere is nurses’ appreciation of working environment, and its characteristics which influence staff nurses, current and future professional benefits which can be directly or indirectly perceived by the members of the organization, as well as impact on staff nurses’ motivation and behavior.

**Aim of the study:**

This study aimed to examine the influence of organizational atmosphere on current and future professional benefits among staff nurses.

**Methods:**

A descriptive cross-sectional study was conducted at Benha University Hospitals, Benha, Egypt. The sample included 2600 staff nurses. Data were collected using two validated instruments, namely: the Organizational Atmosphere Survey and the Nurses' Perceived Professional Benefits Questionnaire (NPPBQ).

**Results:**

The findings revealed that the organizational atmosphere positively predicts employees’ expectations of future professional benefits (β = 0.32, p < 0.001) indicating that better organizational atmospheres are associated with more optimistic professional outlooks. The current perceived benefits had an even moderate effect (β = 0.45, p < 0.001), suggesting that current satisfaction is a key factor in shaping expectations about future development.

**Conclusion:**

The study highlights the importance of an organizational atmosphere to improve current and future professional benefits implication among staff nurses which answers the research hypothesis. This study's findings raise several important implications for nursing practice. At the practice level, there is a necessity for hospital administrators to build the organizational atmosphere through appropriate staffing, supportive leaders, fair compensation, and interprofessional interaction can improve staff nurses' satisfaction now and confidence for future practice.

**Trial registration number:**

Not applicable.

**Clinical trial number:**

Not applicable.

## Introduction

The third millennium has introduced complex challenges to healthcare systems worldwide, including increased competition, rapid organizational change, quality management demands, workforce shortages, ethical concerns, and economic and political pressures [1, 2]. Within this context, the nursing work environment has emerged as a critical determinant of both patient outcomes and nurses’ professional well-being [3]. A supportive and well-structured organizational atmosphere is essential for optimizing healthcare performance and sustaining nursing practice [4, 5].

Staff nurses are particularly vulnerable to adverse working conditions, which have been associated with burnout, job dissatisfaction, anxiety, depression, and increased turnover intentions [6]. High workloads, extended working hours, and inadequate organizational support further exacerbate these challenges [7, 8]. Conversely, nurses’ conscientiousness and sense of responsibility toward their organizations play a pivotal role in enhancing care quality and organizational effectiveness, underscoring the importance of a healthy work environment [9].

Organizational atmosphere refers to nurses’ shared perceptions of the physical, social, and psychological characteristics of their workplace, which shape attitudes, behaviors, and professional engagement [10]. These perceptions are dynamic and influenced by leadership styles, organizational culture, communication patterns, managerial support, and ethical practices [11]. Evidence suggests that a positive organizational atmosphere promotes psychological well-being, job satisfaction, commitment, and professional performance [12, 13]. However, findings from various studies remain inconsistent, with some reporting generally positive perceptions of organizational climate [14, 15], while others indicate moderate or less favorable views, highlighting contextual variability and the need for further investigation [16, 17].

In recent years, increasing attention has been directed toward nurses’ perceived professional benefits, defined as nurses’ subjective evaluations of the rewards, advantages, and personal growth opportunities derived from their profession [18, 19]. Perceived professional benefits contribute significantly to professional identity, job satisfaction, psychological availability, and retention. Importantly, these benefits encompass both current perceived benefits and future anticipated professional benefits, which reflect expectations of career development, recognition, and long-term professional fulfillment [5, 20].

When nurses perceive a discrepancy between high current benefits and low future anticipated benefits, feelings of relative deprivation may arise, leading to psychological strain, emotional exhaustion, and reduced work engagement [21]. In contrast, strong expectations of future professional benefits can enhance nurses’ psychological resources, motivation, and commitment. Despite their importance, empirical studies examining both current and future professional benefits among nurses remain limited, particularly in relation to organizational atmosphere [22, 23].

Existing literature has primarily focused on individual or contextual predictors of perceived professional benefits, such as mindfulness, professional experiences, and demographic characteristics [24]. However, the combined influence of organizational atmosphere on nurses’ current and future professional benefits has received insufficient scholarly attention, especially within low- and middle-income countries such as Egypt [14, 25]. This gap is notable given the global nursing shortage, increasing healthcare demands, and the critical need to improve nurse retention and organizational sustainability [26].

Therefore, the present study aims to examine the influence of organizational atmosphere on current and future perceived professional benefits among staff nurses. By addressing this gap, the study seeks to contribute empirical evidence to nursing administration literature and provide insights to support organizational strategies that enhance nurses’ professional well-being, retention, and the overall quality of healthcare delivery.

## Theoretical framework

This study is theoretically grounded in an integrated framework that combines Organizational Climate Theory, Herzberg’s Two-Factor Theory of Motivation, and Social Exchange Theory to explain how organizational atmosphere influences staff nurses’ current and future perceived professional benefits.

Organizational Climate Theory provides the primary foundation of this study by emphasizing that staff nurses’ shared perceptions of their work environment shape their attitudes, behaviors, and professional experiences. Elements such as leadership style, communication, managerial support, fairness of policies, and professional recognition form the organizational atmosphere through which nurses interpret their work conditions. A positive organizational atmosphere is expected to foster favorable evaluations of both immediate work-related rewards and long-term professional prospects.

To further clarify the mechanisms through which organizational atmosphere affects professional benefits, Herzberg’s Two-Factor Theory is incorporated. According to this theory, hygiene factors (e.g., salary, job security, working conditions) primarily prevent dissatisfaction and are closely aligned with nurses’ perceptions of current professional benefits. In contrast, motivator factors (e.g., professional recognition, opportunities for advancement, continuing education, and career growth) enhance motivation and fulfillment and are more moderate related to future anticipated professional benefits. Within this study, organizational atmosphere is assumed to shape both hygiene and motivator factors, thereby influencing nurses’ evaluations of present rewards as well as expectations of future professional development.

Social Exchange Theory further strengthens the conceptual model by explaining the reciprocal relationship between nurses and their organizations. When nurses perceive that their organization invests in a supportive and fair work environment, they are more likely to reciprocate with positive attitudes, increased engagement, and organizational commitment. This reciprocal exchange enhances nurses’ perceptions of long-term professional benefits, as they expect sustained support, career opportunities, and professional security from the organization.

Integrating these three theories, the proposed conceptual model posits that organizational atmosphere serves as a fundamental organizational input that directly influences staff nurses’ current perceived professional benefits (such as remuneration, recognition, and job-related rewards) and future anticipated professional benefits (such as promotion opportunities, continuing education, and long-term career development). The model further assumes that job satisfaction and work engagement function as key psychological mechanisms through which organizational atmosphere translates into enhanced professional benefit perceptions. Specifically, a positive organizational atmosphere is expected to increase job satisfaction and work engagement, which in turn strengthen nurses’ evaluations of both current and future professional benefits.

**Fig. 1:**
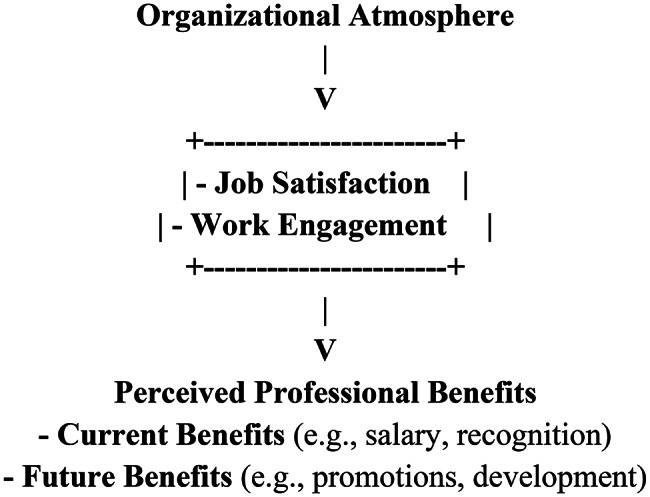
Conceptual model

Based on this integrated theoretical framework, the present study aims to examine the influence of organizational atmosphere on current and future perceived professional benefits among staff nurses, as illustrated in Fig. 1.

## Methods

### Study aim

This study aimed to examine the influence of organizational atmosphere on current and future professional benefits among staff nurses.

### Study hypothesis

#### H_1_

Staff nurses have a measurable perception of the organizational atmosphere within their workplace.

#### H_2_

Staff nurses experience identifiable levels of current and future professional benefits.

#### H_3_

A positive organizational atmosphere is significantly associated with higher perception levels of current and future professional benefits among staff nurses.

### Study setting and design

This descriptive cross-sectional study was conducted in Benha University Hospitals, which are healthcare hospitals affiliated with Benha University in Egypt, total bed capacity was (600 beds), and it is composed of three buildings; Surgical building includes 10 units (160 beds), Medical building includes 24 units (430 beds), and Ophthalmology building consists of 2 units (10 beds).

### Sampling and participants

A convenience sample of 2,600 staff nurses was recruited from various clinical departments at Banha University Hospitals using a non-probability sampling technique. This sampling approach was selected due to its feasibility and practicality in accessing a large and diverse nursing workforce across multiple departments within a limited time frame. Given the demanding nature of nursing schedules and institutional constraints, probability sampling methods were not feasible for this study.

Although convenience sampling does not allow for full population representativeness, the large sample size and inclusion of nurses from different units and specialties were intended to enhance the diversity of perspectives and reduce potential sampling bias. The sample is therefore considered reflective of the accessible nursing workforce at Banha University Hospitals rather than the entire target population.

The adequacy of the sample size was further supported by a post hoc power analysis. With a sample size of 2,600 participants, the study had sufficient power (≥ 0.95) to detect small-to-moderate effect sizes (f² = 0.02–0.15) at a significance level of α = 0.05, which is considered adequate for multivariate analyses and structural equation modeling.” and ensures stable parameter estimation, thereby strengthening the robustness of the study findings.

**Inclusion criteria**: required participants to be currently employed staff nurses with at least 12 months of work experience at Banha University Hospitals, ensuring sufficient exposure to and familiarity with the organizational atmosphere. **Exclusion criteria**: Staff nurses with less than one year of experience, those on extended leave, or those absent during the data collection period.

### Study instruments

An online survey was distributed to 2600 staff nurses in various units at Benha University Hospitals. Staff nurses were shared in filling the survey of the study via WhatsApp which contained a link to the survey created using Google Forms. The message sent via WhatsApp included details about the study objectives and requested approval for participation. Staff nurses were required to click the confirmation button to give their consent and were informed that completing the survey also meant their consent. The survey was organized into three sections.

**Section**,** one** collected staff nurse’s personal /job characteristics, created by researchers. It includes variables such as age, sex, marital status, educational qualification, years of experience and workplace income. The personal and professional variables were chosen with great care to represent the participants.

**The second section** employed an organizational atmosphere (climate) survey developed by Martein [27] and adopted from Hamed et al. [28] a validated instrument designed to assess organizational climate as perceived by staff nurses. The survey comprises multiple dimensions, including Work facilitation, Concern for staff nurses, Team building. Decision making, Nursing participation. Communication, Customer service, Hospital quality, Nurse/physician relationships and Compensation. Staff nurses responded using a five-point Likert scale (always = 5, occasionally = 4, uncertain = 3, rarely = 2 and never = 1), The study subject had low perception level of organization climate if the total percent score was less than 60%, moderated if it ranged from 60% to less than 75% and high if it ranged from > 75% or more accordance with Hamed et al., whose reliability was completely compatible reporting a high Cronbach’s alpha of 0.85 [28]. This study assessed the internal consistency reliability, which was confirmed with 0.911. The Cronbach’s alpha value for various dimensions ranged 0.810 to 0.872 [29].

**The third section** of the research instruments employed (NPPBQ) was developed by Hu [30]. It was used to assess the gains and benefits brought about by the nursing profession and helps in determining whether nursing can enhance overall personal growth. The researchers employed both back-to-back-translation approaches to translate the original questionnaire, also to ensure validity and cultural appropriateness in the framework of (NPPBQ). The instruments were assessed by a panel of specialists in nursing administration and management and experts in knowledge management within healthcare settings. Their contribution helped improve and adapt several questions to better reflect the research’s focus on (NPPBQ). The questionnaire comprises positive occupational perception (3 elements), recognition from family members, relatives, and friends (3 elements), sense of belonging to a team (3 elements), good nurse-patient relationship (4 elements), and self-growth (4 elements), with a total of 17 elements. Each element was rated on a 5- point scale from 1 (strongly disagree) to 5 (strongly agree). Scores were calculated by averaging the responses for each dimension, The study subject had low perception level if the total percent score was less than 60%, moderated if it ranged from 60% to less than 75% and high if it ranged from > 75% or more accordance with Hu [30]. Wan et al. [31] whose reliability was completely compatible reporting a high Cronbach’s alpha of 0.94. This study assessed the internal consistency reliability, which was confirmed with 0.911. The Cronbach’s alpha values for various dimensions ranged between 0.785 and 0.823 [29].

### Instrument validity

The face and content validity of the study instruments were determined by a panel of five experts from the Nursing Administration Departments affiliated with Ain Shams and Cairo Universities in Cairo, Egypt. Prior to expert review, the Nursing Professional Perceived Benefits Questionnaire (NPPBQ) was translated into Arabic using a standardized forward–backward translation procedure to ensure linguistic and conceptual equivalence. Initially, the original English version was translated into Arabic by two independent bilingual nursing experts. The translated versions were reviewed and synthesized into a single preliminary Arabic version.

Subsequently, the preliminary Arabic version was back-translated into English by two independent bilingual translators who were blinded to the original instrument. The back-translated version was compared with the original questionnaire to identify and resolve any discrepancies, ensuring semantic and conceptual consistency.

The Arabic version of the NPPBQ was then reviewed by a panel of five experts in nursing administration and research methodology to assess content validity, clarity, and cultural relevance. Minor modifications were made based on their feedback. Construct validity of the instruments were further assessed using confirmatory factor analysis (CFA), which supported the original factor structure of the instrument.For organizational climate survey, Confirmatory Factor Analysis (CFA) was performed to assess construct validity. The CFA results indicated good model fit: χ²/df = 2.87, RMSEA = 0.048, CFI = 0.962, TLI = 0.955. All factor loadings were significant and ranged from 0.68 to 0.87, supporting the dimensional structure of the organizational atmosphere survey. While for the Nursing Professional Perceived Benefits Questionnaire (NPPBQ), CFA was conducted for the NPPBQ, showing an acceptable model fit: χ²/df = 3.12, RMSEA = 0.051, CFI = 0.958, TLI = 0.950. Factor loadings for individual items ranged from 0.65 to 0.85, confirming the validity of the five-dimensional structure. These findings indicate that the Arabic version of the NPPBQ is a valid and reliable tool for assessing perceived professional benefits among staff nurses.

### Ethical consideration

Researchers confirm that this study was conducted in accordance with the ethical principles outlined in the Declaration of Helsinki. Ethical approval to conduct the study was obtained from the Research Ethics Committee (REC) of the Faculty of Nursing, Benha University, Egypt (REC-NA-P79) in July 2025. Before data collection, the objectives of the study were clearly explained to all participating staff nurses. It was emphasized that all collected data would be used strictly for scientific research purposes. Participants were also informed of their right to voluntary participation, as well as their right to refuse participation or withdraw from the study at any time without facing any negative consequences. To ensure confidentiality and data protection, all responses were anonymized using coded questionnaires, and the data were securely stored in a manner accessible only to the research team.

### Pilot study

Pilot testing was conducted on a group of 10% of the study sample who met the inclusion criteria but were not included in the final analysis. The pilot study aimed to evaluate the clarity, feasibility, and internal consistency of the translated instrument. On average, the tools took approximately 20–25 min to be completed. The data collected from the pilot study were analyzed. The results demonstrated satisfactory reliability, with Cronbach’s alpha coefficients exceeding 0.90 for the overall scale and its subscales, supporting the reliability of the Arabic version.

### Procedures

The study commenced after obtaining ethical approval from the Scientific Research Ethical Committee at the Faculty of Nursing, Benha University. In addition, official permission was secured from relevant healthcare authorities to facilitate the implementation of the study. Prior to data collection, the objectives of the study were thoroughly explained to all participating staff nurses. Voluntary participation was ensured, with clear communication about their rights, including confidentiality and the freedom to withdraw from the study at any point without any consequences. To assess the clarity, feasibility, and reliability of the questionnaire, a pilot study was conducted involving 206 randomly selected staff nurses. The results of the pilot study confirmed the effectiveness of the data collection tools, and no modifications were necessary. This step helped ensure that the instruments were appropriate for the target population.

Several measures were undertaken to reduce potential biases in the study. All questionnaires were coded and anonymized to prevent identification of individual responses. To mitigate social desirability bias, participants were assured of complete anonymity and confidentiality, encouraging honest and unbiased responses. Additionally, to address common method bias (CMB), both design-based and statistical control strategies were employed. Procedurally, the questionnaire was structured to separate different measures, provided with clear instructions, and included distinct response formats to reduce the likelihood of participants providing consistent answers across items. Statistically, Harman’s one-factor test was conducted. All 71 items from the organizational climate, current, and future perceived professional benefits scales were entered into an unrotated principal component analysis. The total variance explained by a single factor was 9.11%, which is well below the commonly accepted threshold of 50% [32], indicating that CMB was not a significant concern in this study. The main phase of data collection spanned a period of two months, beginning in July and concluding at the end of August 2025. During this phase, the online survey was distributed to participating staff nurses across various hospital units. This approach facilitated wide participation and allowed for efficient and timely collection of responses.

### Statistical analysis

Data were analyzed using SPSS version 26 and SmartPLS version 4, each serving distinct analytical purposes. SPSS was used for data screening, descriptive and inferential statistical analyses, while SmartPLS was employed for structural equation modeling (PLS-SEM) to assess the measurement and structural models. Descriptive statistics, including frequencies, percentages, means, and standard deviations, were used to summarize participants’ characteristics and study variables. Pearson’s correlation coefficient was applied to examine bivariate relationships between organizational atmosphere and both current and future perceived professional benefits.

Group comparisons were conducted using non-parametric tests due to non-normal data distribution. The Mann–Whitney U test was used for binary variables (e.g., sex and marital status), while the Kruskal–Wallis H test was applied for variables with more than two categories (e.g., clinical department). Multivariate linear regression analysis was performed using SPSS to examine the predictive effects of organizational atmosphere and current perceived professional benefits on future perceived professional benefits.

Cronbach’s alpha and average variance extracted (AVE) to establish internal consistency reliability and convergent validity. Discriminant validity was assessed using the hetero trait–mono trait (HTMT) ratio. Confirmatory factor analyses were performed to verify the underlying factor structure of the study instruments. All statistical tests were two-tailed. A p-value of < 0.05 was considered statistically significant, while a p-value of < 0.001 was considered highly statistically significant.

## Results

According to Table 1 the study’s results, 68.6% of staff nurses are ranged between 20 and 30 years old, and 73.1% of staff nurses are female. In addition, 61.1% of them were married, 51.0% of them had a technical in nursing degree. Additionally, 53.2% of them had 1–5 years of experiences, 68.4% of staff nurses working in medicine and critical care units, and 66.9% of staff nurses reported inadequate income.

**Table 1:** Personal and job characteristics of studied staff nurses (*n* = 2600)

Personal and job characteristics		Frequency	Percentage %
Age (Years)	20–30 Years	1784	68.6%
	More 30–40 Years	635	24.4%
	More Than 40 Years	181	7%
Sex	Female	1900	**73.1%**
	Male	700	26.9%
Marital Status	Married	1619	**61.1%**
	Unmarried	981	37.0%
Educational Qualification	Nursing Diploma Degree	485	18.3%
	Nursing Technical Degree	1352	**51.0%**
	Bachelor Nursing Degree	549	20.7%
	High studies Degree	214	8.1%
Years of Experience	1–5 Year	1385	**53.2%**
	More 5–10 Years	701	27%
	More 10–15 Years	241	9.3%
	More 15 Years	273	10.5%
Workplace	Medicine and critical care	1778	**68.4%**
	Ophthalmology	270	10.4%
	Surgical	552	21.2%
Income	Adequate	826	31.2%
	Inadequate	1774	66.9%

**Fig. 2:**
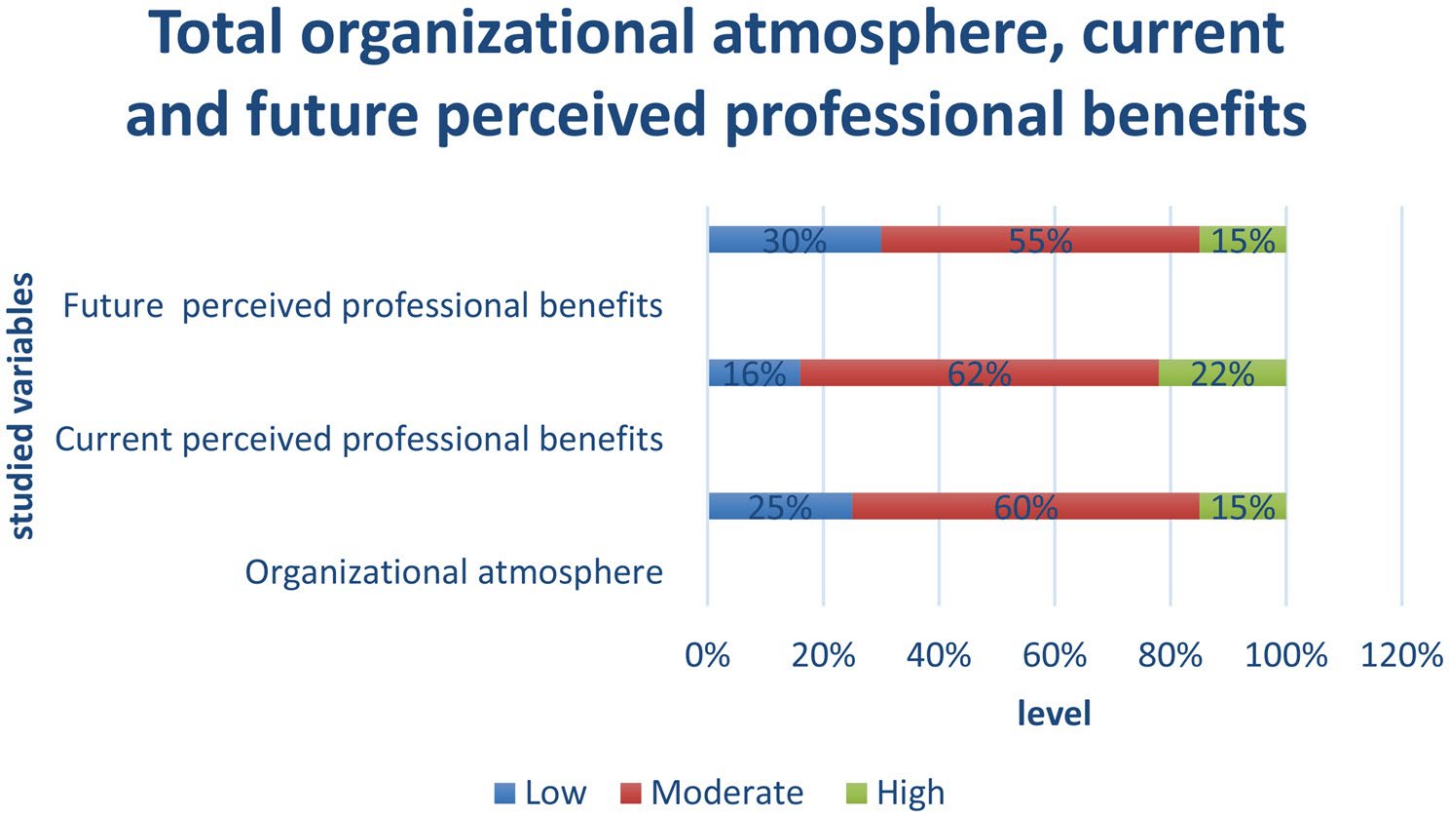
Total organizational atmosphere, current, future professional benefits perception level among staff nurses (*n* = 2600)

Figure 2, For organizational atmosphere, shows that one quarter (25%) of staff nurses reported a low perception level. Over half (60%) exhibited a moderate level, whereas minority 15%) demonstrated a high perception level of organizational atmosphere. For current perceived professional benefits, the result reveals that (16%) of staff nurses reported a low perception level. Over half (62%) exhibited a moderate level, whereas more than one fifth (22%) demonstrated a high perception level of current perceived professional benefits. While for future perceived professional benefits, demonstrates that (30%) of staff nurses reported a low perception level. half (55%) exhibited a moderate level, whereas minority (15%) researchsated a high perception level of future perceived professional benefits.

Table 2 reveals that there was a significant moderate positive correlations (**p < 0.01) between various organizational atmosphere dimensions and both current and future professional benefits among staff nurses.

**Table 2:** Correlation matrix between organizational atmosphere dimensions and current and future professional benefits

Organizational Atmosphere Dimension	Dimensions of current professional benefits				Future professional benefits
	Positive occupational perception	Good nurse-patient relationship	Recognition from family, relatives, and friends	Sense of belonging to a team	
Work facilitation	0.721**	0.614**	0.680**	0.661**	0.649**
Concern for nurse	0.439**	0.353**	0.624**	0.530**	0.567**
Team building	0.688**	0.501**	0.572**	0.661**	0.681**
Decision making	0.609**	0.678**	0.561**	0.542**	0.652**
Nursing Participation	0.543**	0.723**	0.734**	0.653**	0.559**
Communication	0.511**	0.656**	0.475**	0.521**	0.566**
Customer services	0.450**	0.543**	0.501**	0.707**	0.400**
Hospital quality	0.457**	0.389**	0.651**	0.698**	0.519**
Nurse\physician relationship	0.534**	0.811**	0.489**	0.421**	0.523**
Compensation	0.555**	0.602**	0.601**	0.402**	0.566**

Table 3 reveals that the **organizational atmosphere** positively predicts employees’ expectations of future professional benefits (β = 0.32, *p* < 0.001), indicating that better organizational atmosphere are associated with more optimistic professional outlooks. The **current perceived benefits** had an even moderate effect (β = 0.45, *p* < 0.001), suggesting that current satisfaction is a key factor in shaping expectations about future development.

**Table 3:** Multivariate regression analysis between organizational atmosphere, current and future professional benefits

Predictor	B	SE	β (Beta)	T	*p*-value
(Constant)	0.84	0.07	—	12.00	< 0.001**
Organizational Atmosphere	0.38	0.05	0.32	7.60	< 0.001**
Current and future Professional Benefit	0.51	0.05	0.45	10.20	< 0.001**
**Model Summary**					
R²	0.184				
F(2, 2597)	124.56				< 0.001**

**Constant** future perceived professional benefits

Note: B = unstandardized coefficient; SE = standard error; β = standardized beta coefficient; *p* < 0.001 indicates statistical significance

Table 4 demonstrates statistically significant differences by sex in organizational atmosphere, current professional benefits, and future professional benefits (*p* < 0.01). Female nurses reported significantly higher perceptions of organizational atmosphere as well as greater current and future professional benefits compared with male nurses, although the magnitude of these differences ranged from small to moderate (*r* = 0.142–0.161). With respect to educational qualification, differences in current professional benefits approached statistical significance (*p* = 0.050), indicating that nurses with higher educational levels tended to report slightly more favourable perceptions of current professional benefits than those with lower qualifications. No significant differences were found according to marital status for any of the studied variables. Regarding workplace setting, a statistically significant difference was observed only for future professional benefits (H = 12.122, *p* = 0.020, η² = 0.026). Nurses working in different workplace settings reported varying levels of perceived future professional benefits, with some settings offering more optimistic career-related expectations than others. The small effect size indicates that workplace context contributes modestly to these perceptions but does not solely determine them.

**Table 4:** Group differences in organizational atmosphere, current and future professional benefits

Personal and job characteristics	Dependent variable	Z/H Statistics	*P*-value (Asymp. Sig. 2-tailed)	Effect Size (*r*/η²)
Sex	Organizational atmosphere	-2.626	0.007*	0.142ꜛ
	Current professional benefits	-2.909	0.003*	0.161ꜛ
	Future professional benefits	-2.689	0.006*	0.150ꜛ
Marital Status	Organizational atmosphere	-0.512	0.601*	0.029ꜛ
	Current professional benefits	-0.502	0.615*	0.028ꜛ
	Future professional benefits	-0.149	0.818*	0.002ꜛ
Education qualification	Organizational atmosphere	-0.33	0.711*	0.17ꜛ
	Current professional benefits	-1.902	0.050*	0.102ꜛ
	Future professional benefits	-0.024	0.801*	0.023ꜛ
Workplace	Organizational atmosphere	2.319	0.801**	-0.009ꜛꜛ
	Current professional benefits	7.222	0.201**	0.007ꜛꜛ
	Future professional benefits	12.122	0.020**	0.026ꜛꜛ

*Mann-Whitney U / **Kruskal-Wallis H / ꜛr / ꜛꜛη²

## Discussion

Organizational atmosphere emerges as a key factor influencing how staff nurses view their current professional benefits and anticipate their future opportunities. As essential members of the healthcare team, staff nurses are particularly affected by the quality of their work environment, which can shape their motivation, satisfaction, and career expectations [6]. A positive and supportive atmosphere fosters immediate gains such as recognition, professional learning, and collaboration, while at the same time strengthening long-term prospects related to career progression, stability, and continued development [33].

### Organizational atmosphere

In the current study, nearly two-thirds of staff nurses indicated moderate perception level of organizational atmosphere was moderate. This finding could be explained by the presence of facilitating factors. Many facilitating factors such as positive peer relationships and teamwork promote collegiality and belonging, managerial care and support bolster morale, standardized hospital policies and processes add order and predictability to staff nurses’ work, and many staff nurses feel pride in and experience informal learning when they have the opportunity to practice in a high-acuity clinical environment. These facilitatory factors combined help keep staff nurses’ cognitions above a “low” organizational atmosphere level.

This result aligned with similar findings provided by Rakha et al. [34] on staff nurses’ perceptions of organizational climate in governmental hospitals in Ismailia, Egypt. Shahnavazi et al. [35], as well as El-Ghabor [36] also had similar results reporting that more than half of the staff nurses had rated the organizational climate at a moderate perception level. Internationally, similar results demonstrated moderate ratings such as Muñiz et al. and Xu et al. [37, 38] in China who both found that staff nurses had moderate views of organizational climate. Likewise, locally El-Demerdash & Mostafa, and El-Adly [39, 40] supported reporting moderate to moderate perception level of organizational climate in the medical and surgical departments. Similarly, Giacomo [41], reported positive views of organizational climate were reported among critical and intensive care nurses, stating there was strong bond of spirit and collegiality.

On the other hand, some studies recorded low perceptions. Radwan et al. [42] found that over half of staff nurses perceived the climate was low, arguing that factors like inadequate resources, time pressure, limited involvement in decision-making, and lack of rewards played a role. El-Salam et al. [43] pointed out that staff nurses in specialized hospitals rated the organizational climate as at a low perception level. These contrasts also reinforce the importance of context, resource availability, and management style on how organizational atmosphere is experienced.

### Current and future professional benefits

This study’s findings showed that most staff nurses perceived moderate current and future professional benefits. Interestingly, over half of the respondents reported a moderate perception of current professional benefits, while a minority reported a high perception of current professional benefits. From the researchers’ perspective, the findings indicate that most staff nurses perceive their current and future professional benefits as moderate, suggesting a level of satisfaction that is neither minimal nor optimal. It is noteworthy that more than half of the participants reported only a moderate perception of current professional benefits, which may reflect limitations in career advancement opportunities, recognition, or incentives in their current roles. The fact that only a minority of staff nurses perceived their current professional benefits as high raises concerns about the adequacy of the professional support systems in place. These results underscore the need for organizational strategies aimed at enhancing professional development, recognition, and long-term career benefits, which are essential for improving job satisfaction and retention among nursing staff.

These findings align with results noted in a number of other studies, including Sun et al., Zhan et al., Xu et al., and Ma et al., [38, 44–46] in which their results demonstrate that staff nurses’ perceptions of professional benefits are frequently at a moderate-level. The significance of professional benefits is emphasized by empirical evidence which indicates that professional benefits moderate impact motivation, burnout, and staff retention, especially in the context of the nurse shortage accordance Mao et al., [47]. On the contrary, studies conducted in Magnet hospitals accordance Aiken et al., & Caricati et al., [48, 49] indicated that when staff nurses can participate in decision-making, professional development opportunities, and recognition programs, and an abundance of available resources, nurses tend to report predominantly high professional benefits levels.

Future professional benefits were similar in that slightly more than half of staff nurses viewed them at a moderate level, a small portion of staff nurses scored a high level, and nearly one third viewed them at a low level. This indicates that these staff nurses may perceive future professional benefits as lower than current ones due to uncertainty in career advancement opportunities, potential organizational constraints, and systemic barriers such as limited promotion pathways, budget restrictions, or workload pressures. While current benefits are tangible and directly experienced, future benefits are speculative and subject to organizational policies, market conditions, and healthcare system changes, which may lead staff nurses to adopt a cautious outlook regarding their professional growth. Additionally, a lack of mentorship, professional development programs, or recognition initiatives could further reduce optimism about future gains, even if current benefits are perceived as satisfactory.

As Sun et al., [44] and Savitsky et al. [50] noted, intrinsic motivators such a sense of calling and extrinsic motivators such as respect from the community act as protective factors sustaining moderate levels of hope while also forgoing considerable workloads. Even so, occupational fatigue is an ever-present threat Cho et al. [51] as it is a powerful predictor of physical, behavioral, and emotional well-being. It is necessary therefore, to examine workload and maintain adequate nurse staffing levels Lai et al., [20] to further protect staff nurses’ overall enjoyment of work and long-term disposition toward nursing as a career.

The resemblance between current and future perceptions also demonstrates structural barriers as a shared theme. In the current study, two-thirds of staff nurses noted underprivileged income, and over half had technical degrees, and a few were limited in their career advancement opportunities. These reflect the findings of Sun et al. [44], which found staff nurses in the moderate-benefit group were less likely to hold a formal title, while those in leadership positions indicated a higher professional benefit. Contextual barriers of low autonomy, inadequate pay, and high-stressful work environments limit the number of staff nurses who view themselves as having moderate current or future benefits, regardless of peer support or professional pride.

In contrast, the majority of staff nurses in the Egyptian context may view future benefits more moderately than current ones because future career growth depends on systemic structures such as advancement opportunities, financial support for professional development, and leadership pathways that are currently constrained in the Egyptian context. Economic pressures, limited promotional systems, cultural norms, and aspirations for career change or migration combine to temper nurses’ expectations about future professional benefits Kotp et al., [21].

### Correlation between organizational atmosphere dimensions and current and future professional benefits

This research showed significant positive correlations (*p* < 0.01) between the dimensions of organizational atmosphere and the present and future perceived professional benefits, providing additional evidence that a positive work environment is integral to influencing staff nurses’ experiences. This is in line with research conducted by Xu et al. [38] who demonstrated that perceptions of all dimensions of professional benefits are positively correlated with workplace climate. Work facilitation, as one dimension of organizational atmosphere, provided the moderate correlations with perceptions of occupational perception, recognition, and team belonging. Offering sufficient resources, supportive structure, and effective policies makes staff nurses feel connected and valued by their teams. Managers should seek to fortify manpower and policies to elevate perceptions of benefits Caricati et al. [49], with Xu et al. [38] indicating that fulfilling personal values and workplace learning is equally important to increasing perceptions of accomplishment.

Managerial concern was also reported to be a key factor and significantly correlated with both current and future professional benefits. Supportive leadership empowers, inspires, and retains staff nurses by lessening burnout, increasing job satisfaction, and fostering greater long-term commitment. This is consistent with AbdELhay et al. [52] who described the role of transformational leadership as being centered on achieving retention, and Wang et al. [26] who emphasized that staff nurses’ well-being or welfare improves optimism and reduces turnover. Likewise, Kalhor [53] and further research from McMurray et al., Abou Hashish, and Olson [54–56] showed that positive climates not only promote organizational commitment but also career development, and alignment with organizational mission and goals.

Recognition was moderate associated with participation and decision-making, lending support to the importance staff nurses place on engagement in organizational activities. When staff nurses share in decisions, it amplifies professional fulfillment and job satisfaction AbdELhay et al. [52]. The study also indicated the highest associated correlation based on nurse–physician and nurse–patient relationships, confirming the vital influence interprofessional collaboration has on enhancing one’s professional identity and perceptions of benefit Sun et al. [44].

Ultimately, compensation showed a moderate yet significant relationship with every benefit aspect, primarily concerning recognition. Income provides not only security for material goods but also a social signal of respect and equity. Geven et al. [57] weighted rewards serve to minimize burnout, while salary affords social status and familial acknowledgment accordance Marzanek et al., Antonacci et al. [58, 59] which together further buoy the nurse’s pride and sense of professional duty. Also, Peng et al. [60] demonstrated that fair compensation-a critical principle of respect and fairness-advanced motivation, career development, and job satisfaction; and AbdElhay et al. [52] affirmed its value as a retention mechanism. Collectively, these findings underscore that a positive organizational climate, rooted in facilitation, leadership encouragement, participation, collaboration, and fair compensation creates a virtuous cycle that does not only sustain satisfaction, but optimism and hope for the future.

### Group differences in organizational atmosphere, current and future professional benefits

In this study, the researchers found significant sex differences in terms of organizational atmosphere, current professional benefits, and future professional benefits. As a result, female staff nurses reported higher levels of engagement/awareness of the organization’s environment than did their male counterparts. These findings may indicate that females are more involved with or aware of the culture of the organization than males. In addition, they may also indicate the impact of societal/cultural factors that affect the career expectations of nurses. In Egypt, nursing is typically seen as a female-dominated career; therefore, women have much more opportunity to develop a fully formed professional identity, commitment to their profession, and desire to pursue available advancement opportunities. In contrast, male nurses are likely to be subject to different societal/cultural expectations than female nurses, which influences their perceptions of the professional benefits provided by or through the support of the organization/society, along with the experience of barriers during their career path(s). Additionally, differences in the availability of mentorship and career development programs/experiences in addition to exposure to leadership roles may also contribute to the differences in perceptions of professional benefits and support.

This is consistent with Wang et al. [26] study, in which male staff nurses in their pediatric cohort reported slightly lower benefit perception scores than female staff nurses, which could indicate that gendered expectations and cultural norms influence the amount of experience of belongingness and recognition in professionalism. Sun et al. [44] also indicated that staff nurses who evidenced higher professional benefits to patients and families at a cluster level were predominantly female, indicating that females may experience a better link between their profession and their social identity than their male counterparts.

While differences by educational qualification were not significant in this study, results were approaching significance for current professional benefits (*p* = 0.052), with a trend indicating that staff nurses with bachelor’s or postgraduate degrees reported more favorable perceptions than those with a diploma in a technical field. From the researcher’s perspective, this may indicate that higher educational attainment is associated with greater access to professional opportunities, such as leadership roles, specialized training, or recognition within the organization. While the finding did not reach conventional significance levels, it highlights a potential disparity worth further investigation, particularly in how educational background influences perceptions of professional growth and workplace value.

This trend supports the findings of Sun et al. [44] whose results demonstrated that higher education qualification was associated with membership in profiles characterized by stronger optimism and commitment to the profession. Wang et al. [26] also indicated that education level was positively associated with perceived benefits, especially in the realm of career development opportunities and recognition. The null effect in this sample may be explained by the relative homogeneity in educational qualifications or the organizational context, where opportunities for advancement are limited regardless of educational qualification.

There were no significant differences between marital statuses related to any of the constructs indicating family obligations may not have considerably influenced staff nurses’ views of organizational climate or profession-related advantages. It may also reflect a broader organizational culture where support systems or role expectations are relatively consistent, regardless of marital status. This finding contrasts with some assumptions that marital or family commitments might shape professional perceptions, and it highlights the need to explore other personal or contextual factors that may have a more pronounced impact on staff nurses’ professional experiences.

This is contradictory to Wang et al. [26] who found family support provided a positive perspective towards professional benefit type scores. It could be a contextual difference; in Egypt for example workplace-related factors (i.e., workload, staffing and available resources) may influence professional perspectives more than personal demographic attributes.

Finally, workplace was significantly associated with future professional benefits, in which the Kruskal–Wallis test found significant variation across units. Staff nurses who work in higher acuity departments, specifically medicine and critical care, may see fewer opportunities for sustainable career development due to high workloads and stress, while those who work in lower acuity units report higher optimism toward their professional future. This finding is supported by Sun et al. [44], who articulated the importance of organizational conditions and support systems in developing benefit profiles, and Wang et al. [26] who acknowledged that while not statistically significant in their study, departmental demands and resources could influence benefits. Therefore, the findings overall indicate that sex and organizational context are more important factors that shape staff nurses’ professional benefit perception, while education and marital status have reduced or context-specific effects.

## Conclusion

The study concluded that staff nurses perceived moderate levels of organizational atmosphere, current, and future professional benefits. Also, there was a moderate positive correlation between organizational atmosphere and both current and future benefits, Furthermore, the organizational atmosphere significantly predicts future professional expectations, with current benefits having the effect.

### Nursing implications

This study’s findings raise several important implications for nursing practice. At the practice level, there is a necessity for hospital administrators to build adequate effective organizational atmosphere through appropriate staffing, supportive leaders, fair compensation, and interprofessional interaction which can improve staff nurses’ satisfaction now and confidence for future practice. At the management level, fostering the ability for shared decision-making, effective communication, and planned team-building activities can provide both recognition and belonging, and heighten career optimism for staff nurses. At the policy level, Hospital policymakers should create a career pathway and professional development opportunities is critical to building professional benefits for all nurses categories, especially staff nurses who have technical degrees have limited career mobility. The implications align with Tiittanen et al. [61] recommendations focused on supportive work environments to help sustain workforce motivation and retention efforts. Implementing these various levels of implications can prevent burnout, promote retention, and improve the quality of patient care while enhancing the effectiveness and sustainability of the healthcare organization. Finally, future nursing research should conduct context-specific interventions that address structural barriers in low- and middle-income countries, to continue enhancing perceptions of professional benefits and workforce stability.

### Study limitations

This study has several limitations that should be considered when interpreting the findings. First, data were collected using self-report questionnaires, which may introduce response and social desirability bias. Second, the study employed a cross-sectional design, limiting the ability to draw causal inferences between variables. Third, a convenience sampling method was used, which may affect the generalizability of the results to the broader nursing population. Fourth, online data collection could have excluded participants with limited access to digital platforms, potentially introducing selection bias. Finally, since all variables were measured using the same method at a single point in time, there is a risk of common method bias influencing the observed relationships. Future research using longitudinal designs, randomized sampling, and multi-method assessments is recommended to address these limitations.

## Data Availability

The data that support the findings of this study are available from the corresponding author upon reasonable request.
